# Severe cutaneous adverse reactions to anti-osteoporosis drugs: a real-world pharmacovigilance study using the FDA Adverse Event Reporting System database and a review of published cases

**DOI:** 10.3389/fphar.2025.1707885

**Published:** 2025-11-26

**Authors:** Jun-Wei Li, Xin-Dong Lei, Bing Dai

**Affiliations:** 1 The First Hospital of Hunan University of Chinese Medicine, Changsha, Hunan, China; 2 Sichuan Academy of Chinese Medicine Sciences, Chengdu, Sichuan, China

**Keywords:** anti-osteoporosis drugs, severe cutaneous adverse reactions, pharmacovigilance, case review, adverse event reporting system

## Abstract

**Objective:**

This study analyzed severe cutaneous adverse reactions (SCARs) linked to anti-osteoporosis drugs using FDA Adverse Event Reporting System (FAERS) data and characterized implicated drugs and clinical features through a literature review.

**Methods:**

A retrospective disproportionality analysis of SCAR reports from FAERS (2004–2024) utilized signal detection metrics, including reporting odds ratio (ROR), proportional reporting ratio (PRR), and Bayesian confidence propagation neural network (BCPNN). A structured literature search across PubMed, Web of Science, and Scopus gathered case reports of SCARs induced by anti-osteoporosis drugs.

**Results:**

Of 77,789 SCAR reports, 399 (0.51%) involved anti-osteoporosis drugs, mainly affecting female patients (76.25%) with a median age of 69 years. Denosumab (24%), alendronate (23.25%), and zoledronic acid (17.13%) were most frequently reported. Significant signals included risedronic acid with erythema multiforme [ROR = 9.06; PRR = 9.03; information component (IC) = 3.17], zoledronic acid with cutaneous vasculitis (ROR = 3.15; PRR = 3.15; IC = 1.65), and alendronic acid with Stevens–Johnson syndrome (SJS) (ROR = 4.03; PRR = 4.02; IC = 2.00). The literature review (33 cases) confirmed a median symptom onset of 22 days, with treatments often involving corticosteroids and supportive care.

**Conclusion:**

Anti-osteoporosis drugs, notably bisphosphonates and strontium ranelate, are rarely linked to SCARs but may cause serious consequences. Increased clinical awareness, pre-treatment risk evaluation, and vigilant monitoring are essential for at-risk patients.

## 1 Introduction

Severe cutaneous adverse reactions (SCARs) are a group of rare but potentially fatal T cell-mediated type IV hypersensitivity reactions, encompassing Stevens–Johnson syndrome (SJS), toxic epidermal necrolysis (TEN), drug reaction with eosinophilia and systemic symptoms (DRESS), and acute generalized exanthematous pustulosis (AGEP) (Temp et al., 2022). Despite their low overall incidence—ranging from 0.4 to 1.2 cases per million annually—the associated morbidity and mortality are substantial, particularly in TEN, where fatality rates may reach 48%, compared to 2%–6% in DRESS and approximately 4% in SJS (2, 3). Early diagnosis and prompt withdrawal of the suspected causative drug are critical determinants of clinical outcomes. Although extensive pharmacovigilance research has elucidated SCARs associated with antiepileptic drugs (Wei et al., 2023), immune checkpoint inhibitors (Li et al., 2024), and antifungal agents (Shan et al., 2025), limited systematic pharmacovigilance analysis has been conducted on anti-osteoporosis drugs.

Anti-osteoporosis drugs are generally regarded as having an overall good tolerance profile (Varenna et al., 2013). However, rare reports have documented severe cutaneous adverse reactions associated with anti-osteoporosis drugs. In particular, bisphosphonates are linked to SJS and TEN (Barrera et al., 2005), while strontium ranelate is associated with DRESS (Kolitz et al., 2021) and TEN (Yang et al., 2014). Previous reviews, such as Musette et al. (2011), have highlighted rare cutaneous adverse reactions associated with anti-osteoporosis drugs, particularly bisphosphonates and strontium ranelate. These exceptionally rare but severe dermatological toxicities underscore the urgent need for pharmacovigilance studies to evaluate adverse reactions to anti-osteoporosis drugs, particularly rare SCARs, as an increasing number of novel agents enter clinical use.

Real-world pharmacovigilance using spontaneous reporting systems, such as the FDA Adverse Event Reporting System (FAERS), provides a valuable tool for detecting potential safety signals related to rare adverse drug reactions, despite limitations such as voluntary reporting, potential biases, incomplete data, and the lack of causality assessment. These constraints necessitate cautious interpretation to avoid the misattribution of false-positive signals (Morris et al., 2024). Disproportionality analysis tools—such as the reporting odds ratio (ROR) and proportional reporting ratio (PRR)—have proven effective in quantifying drug–event associations and prioritizing high-risk agents (Fusaroli et al., 2024a). This method has successfully characterized SCAR signals across various therapeutic classes, revealing, for instance, that certain antifungals [e.g., fluconazole: ROR 9.50 (Shan et al., 2025)] and immunotherapies [e.g., pembrolizumab: ROR 4.93 (Zhu et al., 2021)] exhibit strong associations with SCARs.

In light of the existing knowledge gaps surrounding anti-osteoporosis drug-induced SCARs, this study aimed to (1) characterize SCARs related to commonly prescribed anti-osteoporosis drugs using FAERS data from 2004 to 2024; (2) compare SCAR signal intensities across drug subclasses; and (3) summarize demographic, clinical, and prognostic patterns through a literature review. The findings will inform risk stratification strategies and contribute to safer, more personalized management of osteoporosis therapy.

## 2 Methods

### 2.1 Data source

The FAERS, a globally recognized spontaneous reporting database, was used in this study. FAERS data are anonymized and updated on a quarterly basis. Raw data were retrieved using the OpenVigil 2.1 platform, a third-party tool designed for standardized data processing, widely used in pharmacovigilance for data extraction, mining, and analysis.

### 2.2 Identification of anti-osteoporosis drugs and adverse events

Anti-osteoporosis drugs were selected based on the World Health Organization’s Anatomical Therapeutic Chemical (ATC) classification system, initially identifying 27 drugs. To address potential confounders such as polypharmacy and comorbidities, drugs were included if they were indicated for osteoporosis treatment and designated as the “primary suspect” in FAERS reports, resulting in the selection of 12 drugs: etidronic acid (M05BA01), pamidronic acid (M05BA03), alendronic acid (M05BA04), ibandronic acid (M05BA06), risedronic acid (M05BA07), zoledronic acid (M05BA08), denosumab (M05BX04), romosozumab (M05BX06), raloxifene (G03XC01), estradiol (G03CA03), teriparatide (H05AA02), and abaloparatide (H05AA04). Exclusion criteria included drugs not primarily indicated for osteoporosis or those reported as secondary suspects or concomitant medications. Adverse events were limited to SCARs, identified using a narrow Standardized MedDRA Query (SMQ) search (MedDRA version 23.1, SMQ code: 20000020), encompassing 18 preferred terms (PTs), including SJS, TEN, DRESS, and AGEP; details are presented in Table 1. Cases lacking sufficient data (e.g., missing drug or event details) or not meeting the SMQ criteria were excluded. The study covers reports from 1 January 2004 to 31 December 2024.

**TABLE 1:** Eighteen narrow-scope PTs in the SMQ classification of SCARs.

PT	MedDRA code
Acute generalized exanthematous pustulosis	10048799
Bullous hemorrhagic dermatosis	10083809
Cutaneous vasculitis	10011686
Dermatitis bullous	10012441
Dermatitis exfoliative	10012455
Dermatitis exfoliative generalized	10012456
Drug reaction with eosinophilia and systemic symptoms	10073508
Epidermal necrosis	10059284
Erythema multiforme	10015218
Erythrodermic atopic dermatitis	10082985
Exfoliative rash	10064579
Oculomucocutaneous syndrome	10030081
SJS–TEN overlap	10083164
Skin necrosis	10040893
Stevens–Johnson syndrome	10042033
Target skin lesion	10081998
Toxic epidermal necrolysis	10044223
Toxic skin eruption	10057970
Severe cutaneous adverse reactions (SMQ)	20000020

### 2.3 Data processing and signal detection criteria

This study adheres to the Reporting of A Disproportionality Analysis for Pharmacovigilance (READUS-PV) guideline to ensure transparent and comprehensive reporting of disproportionality analyses (Fusaroli et al., 2024b). Key elements include the following: (1) a clear definition of the study population and data source (FAERS, 2004–2024, accessed via OpenVigil 2.1); (2) specification of case and non-case selection criteria, including primary suspect drugs and narrow SMQ for SCARs; (3) ensuring reliable detection of significant signals by considering the sample size, with positive signals identified based on the multiple disproportionality metrics according to the following criteria: a minimum of three reported cases (N ≥ 3); ROR ≥2 with the lower bound of the 95% confidence interval (CI) exceeding 1; N ≥ 3, PRR ≥2, and χ^2^ ≥ 4; and information component (IC) > 1 and IC_025_ > 0; and (4) ensuring data integrity and avoiding overestimation of signals by identifying and removing duplicate reports in the FAERS database using a systematic approach. First, multiple versions of the same report (e.g., follow-up reports) were identified using the unique case ID, which includes a suffix indicating follow-up numbers, and only the most recent version of each report was retained. Subsequently, potential duplicates were manually reviewed by cross-referencing key data fields, including patient demographics (age and sex), event date, drug name, adverse event, and reporter country. Reports with identical or highly similar data across these fields were consolidated to retain only one record per unique case. (5) Data were extracted from the dataset, including the year of the report, patient demographics (gender, age, and nationality), and clinical outcomes. Continuous variables were reported as the means ± standard deviations, and categorical variables were expressed as percentages. All signal detection metrics (ROR, PRR, and IC) are reported to two decimal places for consistency, unless specified otherwise. The formulas used for these calculations are presented in Tables 2, 3.

**TABLE 2:** Two-by-two contingency table for disproportionality.

	Drug of interest	Other drug	Total
Adverse event of interest	a	b	a + b
Other adverse events	c	d	c + d
Total	a + c	b + d	a + b + c + d

**TABLE 3:** Summary of major algorithms used for signal detection.

Algorithm	Equation	Criteria
ROR	ROR = (a/b)/(c/d) 95% CI = e^ln(ROR) ± 1.96(1/a+1/b+1/c+1/d)^0.5^	95% CI > 1, N ≥ 2
PRR	PRR = [a/(a + c)]/[b/(b + d)] χ2 = Σ[(O-E)2/E]; [O = a, E=(a + b)(a + c)/(a + b + c + d)]	PRR ≥2, χ2 ≥ 4, N ≥ 3
BCPNN	IC = log2a (a + b + c + d)/[(a + c)(a + b)] IC_025_ = e^ln(IC)−1.96(1/a+1/b+1/c+1/d)^0.5^	IC_025_ > 0

Abbreviations: BCPNN, Bayesian confidence propagation neural network; CI, confidence interval; IC, information component; IC_025_, the lower limit of the 95% two-sided CI of the IC; N, the number of co-occurrences; PRR, proportional reporting ratio; ROR, reporting odds ratio; χ2, chi-squared.

### 2.4 Review of published cases

**FIGURE 1:**
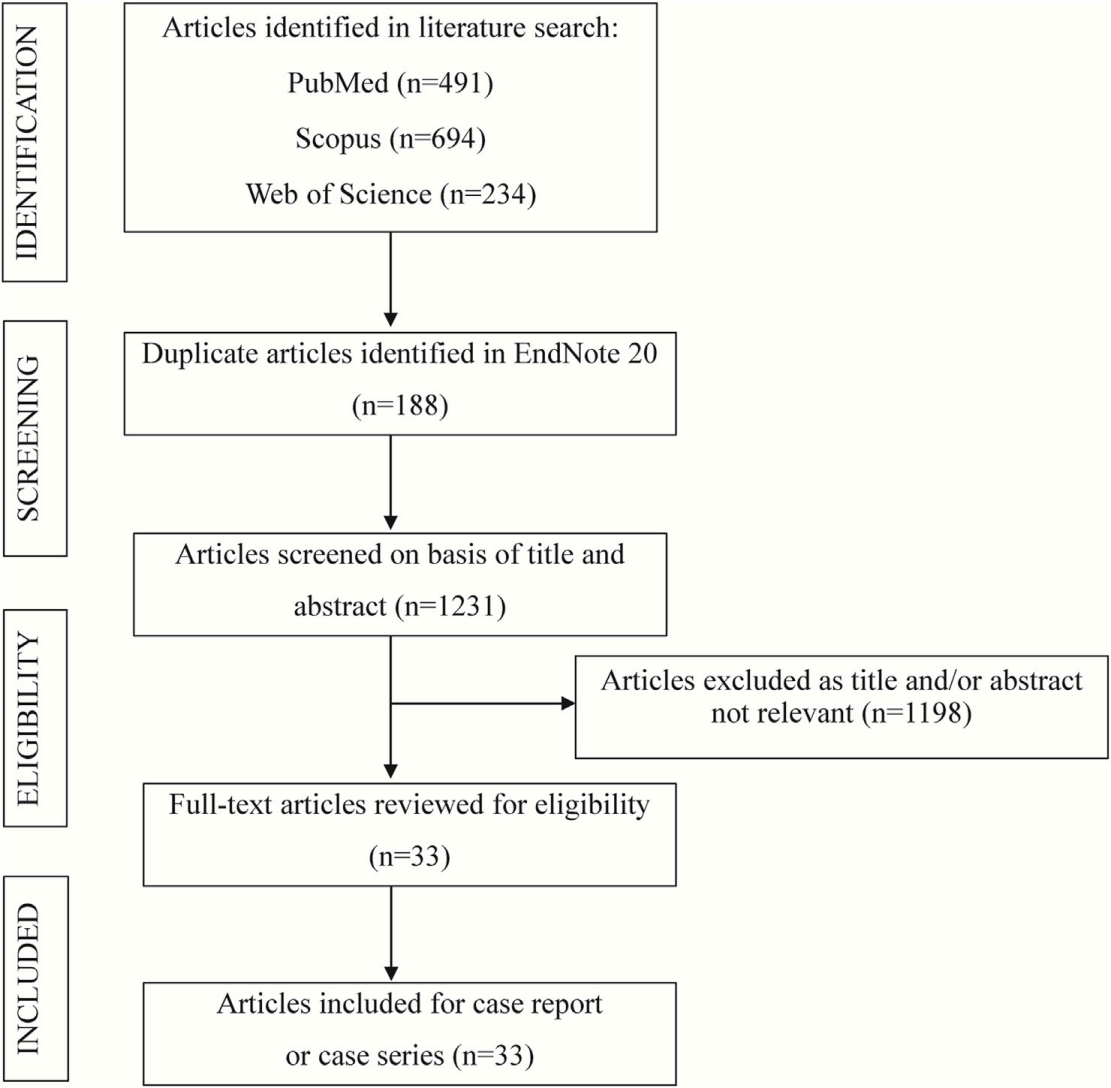
Case reports from the literature search meeting inclusion criteria for the review.

The systematic literature review was conducted in accordance with the Preferred Reporting Items for Systematic Reviews and Meta-Analyses (PRISMA) guidelines to ensure methodological rigor. A structured search was conducted across PubMed, Web of Science, and Scopus from inception to 20 October 2025. Details of the search strategy used in the case review are presented in Table 4. Studies were eligible for inclusion if they satisfied the following criteria: the publication was a case report or case series (Temp et al., 2022); the study described SCARs associated with anti-osteoporosis drugs, identifying 18 SCARs-SMQ preferred terms, including SJS, TEN, DRESS, and AGEP (Hsu et al., 2016); and detailed patient and ADR data were reported, with the full text accessible (Liang et al., 2024). Studies were excluded based on the following criteria: failure to meet the specified study type (Temp et al., 2022); reporting of duplicate cases (Hsu et al., 2016); classification as secondary literature (Liang et al., 2024); unavailability of full text or absence of patient-specific information (Wei et al., 2023); and systematic reviews, meta-analyses, commentaries, clinical guidelines, *in vitro* studies, or animal studies (Li et al., 2024). Study selection was performed independently by two reviewers, with discrepancies resolved through consensus to ensure methodological rigor. For each case, the following variables were extracted: patient demographic characteristics, including country of origin, age, and sex (Temp et al., 2022); details of the anti-osteoporosis drugs associated with cutaneous toxicity, encompassing the generic name, therapeutic indication, clinical presentation of the cutaneous ADR, time to onset of the ADR, histopathological findings from skin biopsies, implemented interventions, clinical outcomes, and time to resolution (Hsu et al., 2016). A PRISMA flow diagram (Figure 1) illustrates the study selection process.

**TABLE 4:** Details of the search strategy used in the case review.

Search strategy item	Details
Keywords	(((((((((((((((((((((((((((etidronic acid) OR (clodronic acid)) OR (pamidronic acid)) OR (alendronic acid)) OR (tiludronic acid)) OR (ibandronic acid)) OR (risedronic acid)) OR (zoledronic acid)) OR (dibotermin alfa)) OR (eptotermin alfa)) OR (ipriflavone)) OR (aluminum chlorohydrate)) OR (strontium ranelate)) OR (denosumab)) OR (burosumab)) OR (romosozumab)) OR (vosoritide)) OR (menatetrenone)) OR (strontium ranelate and colecalciferol)) OR (raloxifene)) OR (bazedoxifene)) OR (estradiol)) OR (parathyroid gland extract)) OR (teriparatide)) OR (parathyroid hormone)) OR (abaloparatide)) OR (palopegteriparatide)) AND (((((((((((((((((((((((“Acute Generalized Exanthematous Pustulosis” [Mesh]) OR (Pustulosis, Exanthematous, Acute Generalized)) OR (Acute Generalised Exanthematous Pustulosis)) OR (Acute Localized Exanthematous Pustulosis)) OR (Pustulosis, Exanthematous, Acute Localized)) OR (bullous haemorrhagic dermatosis)) OR (((((((“Skin Diseases, Vascular” [Mesh]) OR (Skin Disease, Vascular)) OR (Vascular Skin Disease)) OR (Vascular Skin Diseases)) OR (Cutaneous Vasculitis)) OR (Cutaneous Vasculitides)) OR (Vasculitis, Cutaneous))) OR ((((((((((((((((((((((((“Skin Diseases, Vesiculobullous” [Mesh]) OR (Skin Disease, Vesiculobullous)) OR (Vesiculobullous Skin Disease)) OR (Vesiculobullous Dermatoses)) OR (Dermatoses, Vesiculobullous)) OR (Vesiculobullous Skin Diseases)) OR (Vesicular Skin Diseases)) OR (Skin Disease, Vesicular)) OR (Vesicular Skin Disease)) OR (Skin Diseases, Vesicular)) OR (Skin Diseases, Bullous)) OR (Bullous Skin Disease)) OR (Skin Disease, Bullous)) OR (Bullous Dermatoses)) OR (Dermatoses, Bullous)) OR (Bullous Skin Diseases)) OR (Pustular Dermatosis, Subcorneal)) OR (Dermatoses, Subcorneal Pustular)) OR (Dermatosis, Subcorneal Pustular)) OR (Pustular Dermatoses, Subcorneal)) OR (Subcorneal Pustular Dermatoses)) OR (Subcorneal Pustular Dermatosis)) OR (Sneddon-Wilkinson Disease)) OR (Sneddon Wilkinson Disease))) OR ((((((((((((((((“Dermatitis, Exfoliative” [Mesh]) OR (Exfoliative Dermatitides)) OR (Exfoliative Dermatitis)) OR (Dermatitis Exfoliative Generalized)) OR (Exfoliative Generalized, Dermatitis)) OR (Dermatitis Exfoliative Generalised)) OR (Dermatitis Exfoliative Generalized)) OR (Exfoliative Generalised, Dermatitis)) OR (Generalized, Dermatitis Exfoliative)) OR (Dermatitis Exfoliative)) OR (Dermatitis Exfoliative)) OR (Exfoliative, Dermatitis)) OR (Exfoliative, Dermatitis)) OR (Dermatitis Exfoliativa)) OR (Erythroderma)) OR (Erythrodermas))) OR (dermatitis exfoliative generalized)) OR (((((((((((“Drug Hypersensitivity Syndrome” [Mesh]) OR (Drug Hypersensitivity Syndromes)) OR (Hypersensitivity Syndrome, Drug)) OR (Hypersensitivity Syndromes, Drug)) OR (Syndrome, Drug Hypersensitivity)) OR (Syndromes, Drug Hypersensitivity)) OR (Drug Reaction With Eosinophilia And Systemic Symptom)) OR (Drug Reaction with Eosinophilia and Systemic Symptoms Syndrome)) OR (Drug Reaction with Eosinophilia and Systemic Symptoms)) OR (DRESS Syndrome)) OR (DRESS Syndromes))) OR (epidermal necrosis)) OR ((“Erythema Multiforme” [Mesh]) OR (Erythema Multiforme))) OR ((((((((((“Dermatitis, Atopic” [Mesh]) OR (Atopic Dermatitis)) OR (Eczema, Atopic)) OR (Atopic Eczema)) OR (Neurodermatitis, Atopic)) OR (Atopic Neurodermatitis)) OR (Neurodermatitis, Disseminated)) OR (Disseminated Neurodermatitis)) OR (Eczema, Infantile)) OR (Infantile Eczema))) OR (exfoliative rash)) OR (oculomucocutaneous syndrome)) OR (SJS–TEN overlap)) OR (skin necrosis)) OR ((((((((((((((((((((((((((((((((((“Stevens–Johnson Syndrome” [Mesh]) OR (Toxic Epidermal Necrolysis Stevens–Johnson Syndrome Spectrum)) OR (Toxic Epidermal Necrolysis Stevens–Johnson Syndrome)) OR (Toxic Epidermal Necrolysis Stevens Johnson Syndrome Spectrum)) OR (Toxic Epidermal Necrolysis Stevens Johnson Syndrome)) OR (Stevens–Johnson Syndrome Toxic Epidermal Necrolysis)) OR (Stevens Johnson Syndrome Toxic Epidermal Necrolysis Spectrum)) OR (Stevens Johnson Syndrome Toxic Epidermal Necrolysis)) OR (Stevens–Johnson Syndrome Toxic Epidermal Necrolysis Spectrum)) OR (Mycoplasma-Induced Stevens Johnson Syndrome)) OR (Syndrome, Mycoplasma-Induced Stevens–Johnson)) OR (Stevens-Johnson Syndrome, Mycoplasma-Induced)) OR (*Mycoplasma* Induced Stevens Johnson Syndrome)) OR (Mycoplasma-Induced Stevens-Johnson Syndrome)) OR (Stevens-Johnson Syndromes, Drug-Induced)) OR (Stevens–Johnson Syndrome, Drug-Induced)) OR (Drug-Induced Stevens–Johnson Syndromes)) OR (Drug-Induced Stevens–Johnson Syndrome)) OR (Drug Induced Stevens Johnson Syndrome)) OR (Drug-Induced Stevens Johnson Syndrome)) OR (Scalded Skin Syndrome, Nonstaphylococcal)) OR (Nonstaphylococcal Scalded Skin Syndrome)) OR (Syndromes, Lyell’s)) OR (Syndrome, Lyell’s)) OR (Lyell Syndrome)) OR (Lyell’s Syndromes)) OR (Lyell’s Syndrome)) OR (Toxic Epidermal Necrolysis)) OR (Toxic Epidermal Necrolyses)) OR (Necrolysis, Toxic Epidermal)) OR (Necrolyses, Toxic Epidermal)) OR (Epidermal Necrolyses, Toxic)) OR (Epidermal Necrolysis, Toxic)) OR (Stevens Johnson Syndrome))) OR (target skin lesion)) OR (toxic epidermal necrolysis)) OR (toxic skin eruption)) OR (severe cutaneous adverse reaction))
Databases searched	PubMed/MEDLINE, Scopus, and Web of Science
Inclusion criteria	P: patients with osteoporosis or patients requiring bone protection treatment
	I: exposure to anti-osteoporosis drugs
	O: severe cutaneous adverse reactions
Exclusion criteria	Study design: systematic reviews, meta-analyses, commentaries, clinical guidelines, *in vitro* studies, or animal studies
	Studies lacking patient-specific information
Language filter	None applied
Target journals	None applied
Publication period	From the database’s inception to 20 October 2025

## 3 Results

### 3.1 Descriptive analysis of SCAR cases

From 1 January 2004 to 31 December 2024, the FAERS database yielded 77,789 SCAR-related reports, of which 399 (0.51%) were associated with anti-osteoporosis drugs as the primary suspect. Predominantly affecting female patients (76.3%), these cases had a median patient age of 68 years (interquartile range: 21–91 years), with the ≥67-year age group comprising the largest proportion (40.1%). Geographically, North America contributed the highest number of reports (150 cases, 37.6%), followed by Europe (35.6%) and Asia (14.0%), reflecting regional variations in reporting practices. Temporally, SCAR reports exhibited a steady increase, with 146 cases (36.6%) recorded from 2019 to 2024, surpassing earlier periods. Severe outcomes were notable, with hospitalization reported in 29.1% of cases and mortality in 6.8% (27 cases). Alendronate-associated SCARs demonstrated the highest hospitalization rate (10.8%), followed by zoledronate (3.8%) and denosumab (3.5%). Detailed demographic and clinical characteristics are presented in Table 5.

**TABLE 5:** Clinical characteristics of patients treated with anti-osteoporosis drugs.

	Etidronic acid	Pamidronic acid	Alendronic acid	Ibandronic acid	Risedronic acid	Zoledronic acid	Denosumab	Romosozumab	Raloxifene	Estradiol	Teriparatide	Abaloparatide	Total
Total	1	8	95	18	26	69	97	7	7	29	39	3	399
Proportion (%)	0.25	2.01	23.81	4.51	6.52	17.29	24.31	1.75	1.75	7.27	9.77	0.75	100
Patient age, years													
Median	72	78	67	69	56	58	72	70	74	62	74	79	68
Range	72	45–84	21–91	55–82	47–88	42–84	33–90	55–88	60–74	33–75	49–87	60–79	21–91
Patient gender, n (%)													
Male		1 (0.25)	20 (5.01)	1 (0.25)	3 (0.75)	17 (4.26)	11 (2.76)				3 (0.75)		56 (14.04)
Female	1 (0.25)	7 (1.75)	63 (15.79)	16 (4.01)	19 (4.76)	43 (10.78)	78 (19.55)	6 (1.50)	7 (1.75)	24 (6.02)	34 (8.52)	3 (0.75)	301 (75.44)
Not reported			12 (3.01)	1 (0.25)	4 (1.00)	9 (2.56)	8 (2.01)	1 (0.25)		5 (1.25)	2 (0.50)		42 (10.52)
Reporting region													
Africa										3 (0.75)	1 (0.25)		4 (1.00)
Asia			14 (3.51)	3 (0.75)	6 (1.50)	8 (2.01)	12 (3.01)	2 (0.50)	5 (1.25)	2 (0.50)	4 (1.00)		56 (14.04)
Europe	1 (0.25)	4 (1.00)	34 (8.52)	6 (1.50)	14 (3.51)	29 (7.27)	41 (10.28)	4 (1.00)	2 (0.50)	2 (0.50)	5 (1.25)		142 (35.59)
Oceania			1 (0.25)			3 (0.75)	5 (1.25)			2 (0.50)			11 (2.76)
North America		2 (0.50)	37 (9.27)	9 (2.26)	1 (0.25)	27 (6.77)	35 (8.77)	1 (0.25)		20 (5.01)	15 (3.76)	3 (0.75)	150 (37.59)
South America						1 (0.25)	4 (1.00)				2 (0.50)		7 (1.75)
Not reported		2 (0.50)	9 (2.26)		5 (1.25)	1 (0.25)					12 (3.01)		29 (7.27)
Reporting year													
2004–2008	1 (0.25)	6 (1.50)	23 (5.76)	7 (1.75)	7 (1.75)	12 (3.01)			2 (0.50)	5 (1.25)	13 (3.26)		76 (19.05)
2009–2013		2 (0.50)	23 (5.76)	5 (1.25)		18 (4.51)	25 (6.27)			4 (1.00)	12 (3.01)		89 (22.31)
2014–2018			12 (3.01)	4 (1.00)	11 (2.76)	9 (2.26)	34 (8.52)			8 (2.01)	8 (2.01)	2 (0.50)	88 (22.06)
2019–2024			37 (9.27)	2 (0.50)	8 (2.01)	30 (7.52)	38 (9.52)	7 (1.75)	5 (1.25)	12 (3.01)	6 (1.50)	1 (0.25)	146 (36.59)
Outcome events n (%)													
Death	1 (0.25)	1 (0.25)	18 (4.51)			2 (0.50)	3 (0.75)				2 (0.50)		27 (6.77)
Disability			3 (0.75)			2 (0.50)	1 (0.25)						6 (1.50)
Life-threatening		1 (0.25)			1 (0.25)	6 (1.50)	1 (0.25)				1 (0.25)		13 (3.26)
Hospitalization—initial or prolonged		3 (0.75)	43 (10.78)	5 (1.25)	7 (1.75)	15 (3.76)	14 (3.51)	1 (0.25)	4 (1.00)	10 (2.51)	14 (3.51)		116 (29.07)
Required intervention to prevent permanent impairment/damage				1 (0.25)									1 (0.25)
Other serious events		3 (0.75)		12 (3.01)	18 (4.51)	44 (11.03)	78 (19.55)	6 (1.50)	3 (0.75)	19 (4.76)	22 (5.51)	3 (0.75)	236 (59.15)

### 3.2 Identification and distribution of suspected culprit drugs

The analysis encompassed 12 anti-osteoporosis drugs, classified according to the World Health Organization’s Anatomical Therapeutic Chemical (ATC) system: etidronate, pamidronate, alendronate, ibandronate, risedronate, zoledronate, denosumab, romosozumab, raloxifene, estradiol, teriparatide, and abaloparatide. Denosumab (24.3%), alendronate (23.8%), and zoledronate (17.3%) emerged as the most frequently associated agents. Among SCAR subtypes, erythema multiforme (65 cases, 16.3%), skin necrosis (50 cases, 12.5%), cutaneous vasculitis (43 cases, 10.8%), Stevens–Johnson syndrome (42 cases, 10.5%), and bullous dermatitis (39 cases, 9.8%) accounted for the majority of reported PTs, as delineated in Table 4.

### 3.3 SCAR signal detection

**FIGURE 2:**
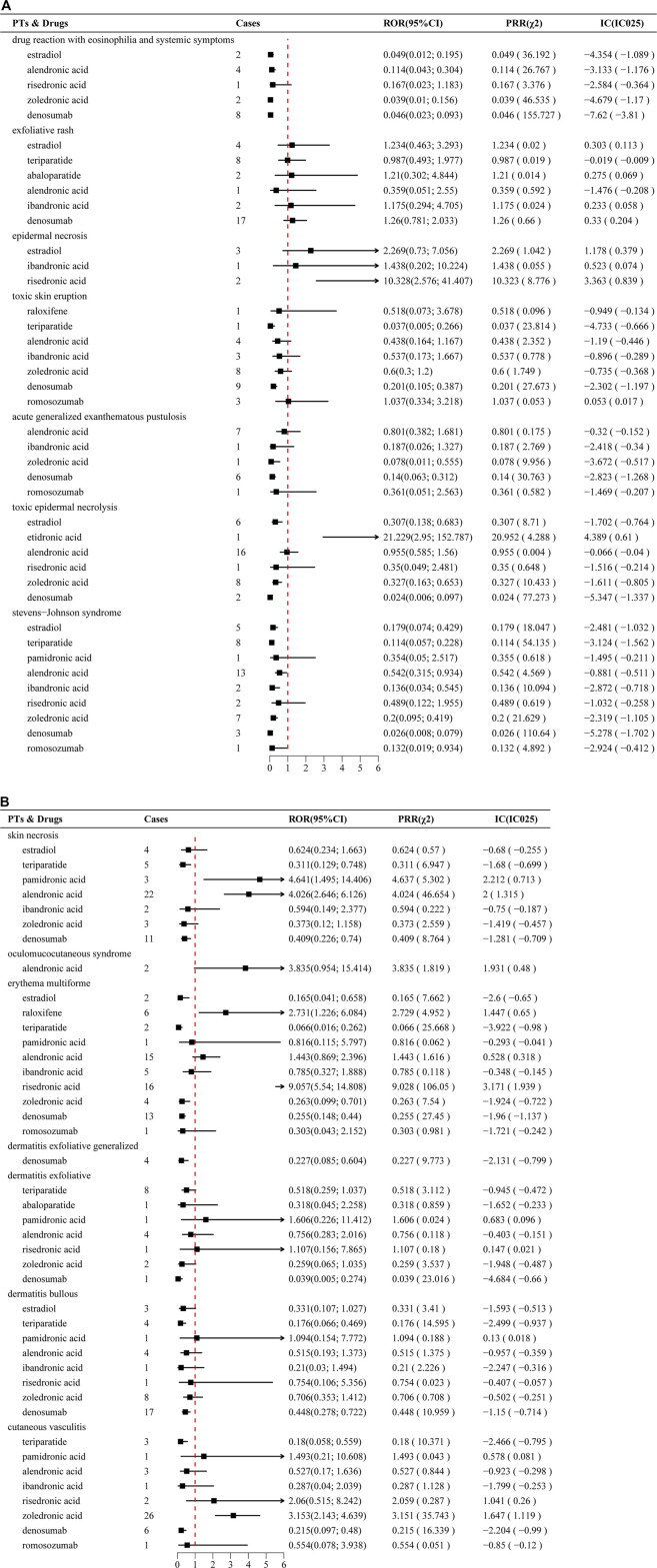
**(A)** Disproportionately reported adverse events of narrow-scope PTs in the SMQ classification of SCARs for anti-osteoporosis drugs in the FAERS database. **(B)** Continuation of Figure 2A.

Disproportionality analyses, using ROR, PRR, and Bayesian confidence propagation neural network (BCPNN), identified significant SCAR signals for several anti-osteoporosis drugs. Signals were deemed statistically significant when meeting predefined thresholds: N ≥ 3, ROR ≥2 (lower 95% CI > 1), PRR ≥2 (χ^2^ ≥ 4), and IC > 1 (IC025 > 0). Notable signals included risedronate with erythema multiforme (n = 16; ROR 9.06, 95% CI 5.54–14.81; PRR 9.03; IC 3.17), zoledronate with cutaneous vasculitis (n = 26; ROR 3.15, 95% CI 2.14–4.64; PRR 3.15; IC 1.65), alendronate with SJS (n = 22; ROR 4.03, 95% CI 2.65–6.13; PRR 4.02; IC 2.00), pamidronate with SJS (n = 3; ROR 4.64, 95% CI 1.50–14.41; PRR 4.64; IC 2.21), and raloxifene with erythema multiforme (n = 6; ROR 2.73, 95% CI 1.23–6.08; PRR 2.73; IC 1.45). No significant signals were detected for denosumab, romosozumab, or teriparatide across evaluated PTs. Comprehensive signal detection results are summarized in Figure 2.

### 3.4 Case report review

A systematic literature review identified 33 case reports involving 35 patients with SCARs attributed to anti-osteoporosis drugs. The median patient age was 67 years (range: 49–85 years), with a pronounced female predominance (94%). From the perspective of drug distribution, among the 35 adverse reactions, strontium ranelate was the most frequently associated agent (n = 16, 50%), followed by bisphosphonates (n = 13; e.g., alendronate and clodronate) and denosumab (n = 3), with raloxifene, teriparatide, and romosozumab each involved in one case. Clinical manifestations included DRESS (11 cases, 10 linked to strontium ranelate and 1 to denosumab), SJS (2 cases, both strontium ranelate), and TEN (1 case, strontium ranelate). The median onset time for SCARs was 22 days (range: 2–180 days). Therapeutic interventions predominantly involved systemic corticosteroids, topical corticosteroids, oral antihistamines, intravenous immunoglobulin, and supportive care. Clinical outcomes were favorable in most cases, with 62% (n = 22) achieving full recovery and 28% (n = 10) showing improvement; however, two strontium ranelate-associated DRESS cases (Drago et al., 2016; Jonville-Béra et al., 2009) and one denosumab-associated c-ANCA vasculitis (Sanchez et al., 2019) resulted in death. It should be noted that this case review has heterogeneity; among the 35 patients, only 21 underwent skin pathological biopsy, while 2 cases underwent Naranjo assessment, and 6 cases included patch testing. It limits the ability to definitively attribute causality in some reports. Detailed case characteristics are presented in Table 6.

**TABLE 6:** Detailed characteristics of the case reports’ patients.

No.	Author/year	Age/sex	Patient nationality	Drug	Adverse reaction	Time to the onset of skin manifestations	Description	Pathology	Management	Time to remission	Outcome	Naranjo score	Patch test
1	Bautista-Villanueva et al. (2021)	55/Male	Spain	Alendronate	Acute localized exanthematous pustulosis	15 days	A flare-up that manifested as erythematous papules with a central pustule on both calves	Subcorneal pustule with neutrophils and eosinophils, dermal edema, and mild spongiosis around the pustule	——	1 week	Recovery	——	Positive
2	Pajus et al. (1993)	70/Male	France	Clodronate	Erythroderma	14 days	Generalized erythematous maculopapular rash without itching, fever at 40 °C, buccal and genital mucosal lesions, and punctate keratitis	Epidermal changes, with a dermal lymphohistiocytic and eosinophilic infiltration	Intramuscular betamethasone and oral H1 antihistamines	Few days	Improvement	——	——
3	Marovt and Marko (2024)	72/Female	Slovenia	Denosumab	Acute generalized exanthematous pustulosis	2 days	Extensive pustules on the trunk and extremities and second, extensive plaques on the extensor sides of the arms, legs, and lower back, affecting approximately 15% of the body surface area	A slightly acanthotic epidermis with focal parakeratosis and the formation of extensive intracorneal neutrophilic pustules. The dermis showed a moderately intense superficial and perivascular mixed-cellular infiltrate with predominantly neutrophilic granulocytes	Corticosteroid	1 week	Recovery	——	——
4	Sanchez et al. (2019)	85/Female	Peruvian	Denosumab	c-ANCA vasculitis	30 days	A small lesion in the ankle with skin rash and telangiectasias	——	Methylprednisolone and then prednisone	4 weeks	Death	——	——
5	Al-Attar et al. (2020)	76/Female	United Kingdom	Denosumab	Drug reaction with eosinophilia and systemic symptoms	180 days	Diffuse pruritic erythematous skin rash and facial swelling	——	Anti-histamines	6 months	Improvement	——	——
6	Song et al. (2019)	61/Female	China	Ibandronate	Erythema multiforme	3 days	Extensive erythema on the upper limbs, lower limbs, and trunk, with coalescing papules and a symmetrical distribution, and the erythema spread to the patient’s face, trunk, and limbs became flushed, and skin lesions coalesced locally, accompanied by a high temperature	——	Antihistamines and glucocorticoids	22 days	Recovery	——	——
7	Weber et al. (2011)	70/Female	France	Ibandronate	Erythematous eruption	25 days	A persisting itchy oedematous erythematous eruption of the lower neck	Leukocytoclastic vasculitis with an important inflammatory perivascular and interstitial infiltrate of lymphocytes and neutrophils in the superficial and medium dermis	Topical steroids	4 days	Recovery	——	——
8	Barthalon et al. (2024)	54/Female	France	Pamidronate	Symmetrical drug-related intertriginous and flexural exanthema	2 days	A pruritic, extensive exanthema involving the main folds (axillary region, breast, and neck)	An eczematous dermatitis	Topical corticosteroids	10 days	Recovery	——	Positive
9	Phillips et al. (1998)	49/Female	Canada	Pamidronate	Urticaria	4 days	Pruritus followed by hives on the dorsum of both feet and buttocks lasting 2 h	——	Diphenhydramine	30 min	Recovery	——	Negative
10	Norimatsu and Norimatsu (2021)	74/Female	Japan	Raloxifene	Erythema multiforme	3 days	A target lesion was scattered around the lower limbs, abdomen, back, and face	Vacuolar degeneration in the base of the epidermis and mild lymphocyte and eosinophil infiltration in the upper dermis	Topical betamethasone butyrate propionate and clobetasol propionate	7 days	Recovery	——	——
11	Belhadjali et al. (2008)	72/Female	Tunisia	Risedronate	Cutaneous vasculitis	21 days	Multiple infiltrated purpuric plaques on both legs	Perivascular neutrophil and eosinophil infiltrates with nuclear debris, accompanied by extravasated red blood cells and fibrin deposition around vessels	——	2 weeks	Recovery	——	——
12	Garcia-Nunez et al. (2021)	53/Female	Spain	Risedronate	Erythema multiforme	3 days	Keratinocyte necrosis, mononuclear cell infiltration, and edema	Differential diagnosis of vasculitis-like syndrome	——	4 days	Recovery	——	Positive
13	Bianchi et al. (2017)	56/Female	Italy	Risedronate	Erythema multiforme-like eruption	A few days	Itchy erythema multiforme-like eruption mainly involving the upper limbs and hands	——	Desoxymethasone and oral oxatomide	2 weeks	Recovery	——	Positive
14	Kolitz et al. (2021)	51/Female	United States of America	Strontium citrate	Drug reaction with eosinophilia and systemic symptoms	42 days	Mucosal erosions, diffuse erythematous papules, confluent plaques on extremities, pseudo-vesicular lesions, and pustular back lesions	Superficial perivascular dermatitis with lymphocyte infiltrate, eosinophils, spongiosis, and basement membrane vacuolation	Topical corticosteroids and methylprednisolone	——	Improvement	——	——
15	Kramkimel et al. (2009)	70/Female	France	Strontium ranelate	Bullous drug reaction with eosinophilia and systemic symptoms	28 days	Initially, a facial rash progressed within a week to edema, trunk blisters, fever, exfoliative erythroderma, cheilitis, conjunctivitis, lymphadenopathy, and multi-organ (liver, lung, and kidney) involvement	——	Topical corticosteroids and antihistamines and systemic corticosteroids	2 weeks	Recovery	——	——
16(1)	Jonville-Béra et al. (2009)	78/Female	France	Strontium ranelate	Drug reaction with eosinophilia and systemic symptoms	10 days	A febrile, diffuse rash	A lymphohistiocytic infiltrate, with eosinophilia in the superficial dermis, and bone medulla was infiltrated with eosinophils (28%)	Prednisone	18 days	Improvement	——	——
16(2)	Jonville-Béra et al. (2009)	69/Female	France	Strontium ranelate	Drug reaction with eosinophilia and systemic symptoms	15 days	Generalization of the rash with fever, facial edema, enanthema, confusion, eosinophilia, and liver damage	——	Methylprednisolone	——	Death	——	——
17	Iyer et al. (2009)	71/Female	United Kingdom	Strontium ranelate	Drug reaction with eosinophilia and systemic symptoms	42 days	A widespread maculopapular rash, fever, and acute renal failure and deranged liver function	——	Intravenous methyl prednisolone and then oral prednisolone	5 weeks	Recovery	——	——
18	Le Merlouette et al. (2011)	77/Female	France	Strontium ranelate	Drug reaction with eosinophilia and systemic symptoms	28 days	Febrile desquamative erythroderma	Moderate spongiosis associated with parakeratotic hyperkeratosis and, at the dermal level, a lymphocytic perivascular infiltrate. Parakeratotic hyperkeratosis and spongiosis, associated with a perivascular lymphocytic infiltrate in the dermis	Prednisone	10 days	Recovery	——	——
19	Kinyó et al. (2011)	69/Female	Hungary	Strontium ranelate	Drug reaction with eosinophilia and systemic symptoms	28 days	Fever and generalized erythroderma	Extensive hydropic degeneration of basal keratinocytes, hyperkeratosis, granular spongiosis, keratinocyte necrosis, and sub-epidermal eosinophilic infiltration	Methylprednisolone and prednisolone	3 m	Improvement	——	——
20	di Meo et al. (2016)	64/Female	Italy	Strontium ranelate	Drug reaction with eosinophilia and systemic symptoms	21 days	Pruritic maculopapular rash involving the trunk, arms, and legs	Keratinocytes with spongiosis, intraepidermal eosinophilic infiltration, suffusion of red blood cells with perivascular granulocytes, and lymphocyte inflammatory infiltrate	Methylprednisolone	3 weeks	Improvement	——	——
21	Drago et al. (2016)	71/Female	Italy	Strontium ranelate	Drug reaction with eosinophilia and systemic symptoms	30 days	A diffuse, itchy maculopapular exanthem. Erythrodermia, and facial edema	Dermal perivascular inflammatory infiltrate of lymphocytes, histiocytes, and scattered eosinophils	Prednisone	——	Death	——	——
22	Moreno-Higueras et al. (2017)	64/Female	Spain	Strontium ranelate	Drug reaction with eosinophilia and systemic symptoms	——	Generalized erythematous rash with papular lesions	——	Prednisone and dexchlorpheniramine	——	Improvement	4	——
23(1)	Smith and Shipley (2010)	83/Female	United Kingdom	Strontium ranelate	Exfoliative dermatitis	41 days	Itchy, raised, and red lesions on back, arms, and chest evolved into widespread scaling erythema over days	Features typical of a drug eruption	Topical steroids and high-dose oral prednisolone	1 month	Improvement	——	——
23(2)	Smith and Shipley (2010)	75/Female	United Kingdom	Strontium ranelate	Exfoliative dermatitis	4 days	Itchy erythematous lesions on back, buttocks, abdomen, and extremities and then progressed to a generalized exfoliative dermatitis	——	Topical steroids and oral prednisolone	21 days	Recovery	——	——
24	Boada et al. (2009)	56/Female	Spain	Strontium ranelate	Generalized cutaneous drug eruption	60 days	Severe generalized exanthema consisting of several erythematous to violaceous tender and confluent-to-plaque papules, pseudovesicular in appearance, with a symmetric distribution on the face, the trunk, and the extremities	Papillary edema and a perivascular mixed infiltrate with eosinophils. In the epidermis, mild spongiosis with necrotic keratinocytes	Oral and topical corticosteroids and oral diphenhydramine	1 month	Recovery	——	Negative
25	Tan et al. (2011)	67/Female	China	Strontium ranelate	Stevens–Johnson syndrome	21 days	Lips with confluent erosions, buccal mucosa, and soft palate ulcerated. Scattered purpuric macules on the chest and palms. Negative Nikolsky’s sign, with small erosion on the left labia majora	Epidermal necrosis with neutrophil aggregates in the stratum corneum, perivascular lymphocytic infiltrate, and sub-epidermal vesiculation noted	Intravenous hydrocortisone and then oral prednisolone, topical triamcinolone oral paste, and an antiseptic mouthwash	——	Recovery	——	——
26	Yang et al. (2014)	70/Female	China Taiwan	Strontium ranelate	Stevens–Johnson syndrome	37 days	Itchy erythematous to purpuric macules and papules on the back spread to the chest, abdomen, and limbs, accompanied by oral mucosal ulceration and genital erosion	Apoptotic keratinocytes, vacuolization of the basal layer, and superficial perivascular lymphocytic infiltration	Methylprednisolone	2 weeks	Improvement	——	——
27	Lee et al. (2009)	72/Female	China	Strontium ranelate	Toxic epidermal necrolysis	9 days	Febrile (40 °C) with targetoid limb lesions, bullae, erosions, conjunctivitis, hemorrhagic cheilitis, orogenital ulcers, 30% epidermal detachment, and positive Nikolsky’s sign	Full thickness epidermal necrosis, dermo-epidermal separation, and moderate lymphocytic infiltrates with scattered eosinophils	Intravenous immunoglobulins	14 days	Recovery	——	——
28	Leis-Dosil et al. (2013)	80/Female	Spain	Teriparatide	Cutaneous vascular calcifications	60 days	Painful necrotic ulcers on the legs and on the areas of the skin, with a livedoid appearance	Ulceration and necrosis of the epidermis, dilatation of the dermal vessels, and circumferential calcification in the walls of small arteries at the dermal–epidermal junction	——	3 weeks	Improvement	——	——
29	Zahedi et al. (2023)	58/Female	United States of America	Zoledronic acid	Cutaneous vasculitis	5 days	Non-blanching, palpable purpura above the ankles and extending to the knees	——	Prednisone	20 days	Recovery	——	——
30	Alghamdi et al. (2024)	64/Female	Saudi Arabia	Zoledronic acid	Delayed inflammatory reaction	2 days	Localized and progressively increasing firm swelling on the face in the jaw and cheeks at the sites of previously injected fillers	——	Oral prednisolone and cetirizine	3 days	Recovery	——	——
31	Nassar and Janani (2021)	53/Female	Morocco	Zoledronic acid	Erythematous macules	2 days	A type of confluent erythematous macules in the trunk and arms and of extended petechial macules in the left thigh and leg	——	Desonide and atoderm anti-itching	6 days	Recovery	——	——
32	Swarnkar et al. (2021)	71/Female	India	Zoledronic acid	Urticarial vasculitis	1 day	Multiple erythematous, edematous papules and plaques with few lesions showing non-scaly purpura over the face, trunk, buttocks, bilateral upper and lower limbs, palms, and soles	Moderately dense perivascular and interstitial infiltrate of neutrophils and some eosinophils, accompanied by nuclear dust and extravasation of red blood cells in the superficial and mid-dermis	Oral antihistamines and topical steroid	1 week	Recovery	5	——
33	Rodriguez Arrieta et al. (2025)	71/Female	Colombian	Romosozumab	Asymmetric erythematous–edematous circinate plaques	1 day	Asymmetric erythematous–edematous circinate plaques with regular and well-defined edges and a perilesional whitish halo associated with local heat on the abdomen	——	Topical corticosteroids (hydrocortisone 0.1% for 7 days) and moisturizing with aluminium acetate lotion	20 days	Recovery	——	——

## 4 Discussion

To our knowledge, this study represents the first comprehensive pharmacovigilance analysis of SCARs associated with anti-osteoporosis drugs using the FAERS database, complemented by a systematic review of published case reports. Using FAERS data from 2004 to 2024, we identified 399 cases of SCARs associated with anti-osteoporosis drugs, representing approximately 0.001% of the 30,390,978 adverse event reports for these drugs in the database. This suggests that SCARs associated with anti-osteoporosis drugs are rare adverse drug reactions. Even though signals derived from FAERS reflect statistical associations rather than definitive causal relationships and SCARs linked to anti-osteoporosis drugs are rare, their clinical significance remains substantial given the potential for severe clinical outcomes.

Demographically, our study found that 76.3% of SCAR cases occurred in female patients, with a median age of 68 years, which corresponds to 94% female patients and a median age of 67 years in the case review. A real-world study also confirmed that the incidence of SCARs was higher among female patients than in male patients, with the majority of cases occurring in the 61–70-year age group (Li et al., 2023). The prevalence of osteoporosis in elderly women represents a key contributing factor. Additionally, age-related decreases in hepatic and renal function, extended drug elimination half-lives, and heightened drug sensitivity collectively increase the propensity for elderly patients to develop adverse drug reactions (Woo et al., 2020). The high hospitalization rate (29.07%) and mortality rate (6.77%) associated with anti-osteoporosis drug-induced SCARs highlight their clinical severity, particularly for alendronic acid (10.78% hospitalization rate). These outcomes may be exacerbated by polypharmacy and comorbidities in elderly patients, which can complicate SCAR management. The case review further revealed that systemic corticosteroids and supportive care were the most common interventions, with recovery or improvement in 90% of cases, although two strontium ranelate-related DRESS cases (Drago et al., 2016; Jonville-Béra et al., 2009) and one denosumab-associated c-ANCA vasculitis (Sanchez et al., 2019) resulted in death (Drago et al., 2016; Kinyó et al., 2011).

Disproportionality analysis revealed significant signals for severe cutaneous toxicity associated with bisphosphonates, underscoring their potential for SCARs. Risedronic acid showed a strong association with erythema multiforme (ROR 9.06), while pamidronic acid and alendronic acid were associated with SJS, with RORs of 4.64 and 4.03, respectively. A pharmacovigilance study involving 13,164 patients in England reported a rare case of risedronate-associated SJS (8). Risedronate-induced erythema multiforme-like eruption (Bianchi et al., 2017) and erythema multiforme (Garcia-Nunez et al., 2021) were reported. Bautista-Villanueva et al. (2021) first reported a case of alendronate-induced acute localized exanthematous pustulosis. de Arruda et al. (2017) reported a case of erythema multiforme associated with alendronic acid. Zoledronic acid-induced erythematous macules (Nassar and Janani, 2021), urticarial vasculitis (Swarnkar et al., 2021), and cutaneous vasculitis (Zahedi et al., 2023) have also been documented. Consistent with prior findings, Norimatsu and Norimatsu (2021) reported the only known case, to date, of a 74-year-old female patient who developed erythema multiforme minor while taking raloxifene. In addition, in a single-center, randomized, double-blind, placebo-controlled study named TEMP, the incidence of etidronate-related hypersensitivity dermatological reactions was 2.7% (1/37) (Kranenburg et al., 2018). These findings emphasize the critical need for vigilance regarding severe cutaneous toxicities associated with bisphosphonate use.

In contrast, no significant signals were observed for denosumab, romosozumab, or teriparatide, potentially due to lower reporting rates or differing immunopathogenic mechanisms. Notably, documented cases have linked teriparatide to multiple pruritic erythematous papules (Chu and Kim, 2016) and cutaneous vascular calcification (Leis-Dosil et al., 2013). Moreover, a recent case was reported of a 71-year-old Colombian woman who developed SCARs, characterized by two asymmetric erythematous–edematous circinate plaques, on the day of romosozumab injection, leading to discontinuation of the treatment (Rodriguez Arrieta et al., 2025). Additionally, denosumab is also one of the most frequently reported drugs. In a large-scale clinical trial involving over 7,800 postmenopausal women with osteoporosis, the incidence of denosumab-related cutaneous adverse events, such as dermatitis, eczema, and rashes, was reported at 10.8% (Cummings et al., 2009). In the FREEDOM trial, serious adverse cutaneous infections, in particular, cellulitis and erysipelas, were observed in 12 (0.3%) participants receiving denosumab (Saag et al., 2018). In our retrospective analysis of clinical cases, we identified instances in which denosumab was associated with the development of AGEP (Marovt and Marko, 2024) and DRESS (Al-Attar et al., 2020). The evidence suggests that although denosumab, romosozumab, and teriparatide are generally safe and well-tolerated biologic agents, these findings highlight the potential for rare yet serious SCARs, warranting careful monitoring and prompt clinical management.

Notably, despite multiple reported cases of strontium ranelate-related SJS (Yang et al., 2014; Tan et al., 2011), TEN (39), DRESS (Kolitz et al., 2021; Drago et al., 2016; Jonville-Béra et al., 2009; Kinyó et al., 2011; Le Merlouette et al., 2011; di Meo et al., 2016; Moreno-Higueras et al., 2017; Iyer et al., 2009; Kramkimel et al., 2009), exfoliative dermatitis (Smith and Shipley, 2010), and generalized cutaneous drug eruption (Boada et al., 2009), among 199 patients with adverse drug reactions to strontium ranelate in France, DRESS accounted for the majority of cutaneous adverse events (19/51 cutaneous AEs) and occurred predominantly in women with a median age of 74 years (range: 58–87 years). The median time to the onset from the initiation of strontium ranelate treatment was 35 days (range: 23–365 days), while one patient died due to fulminant hepatitis associated with DRESS (Jonville-Bera and Autret-Leca, 2011). However, discrepancies were noted, particularly the high prevalence of strontium ranelate-associated SCARs (50% of cases) in the literature, which were absent in FAERS due to its non-approval in the U.S. This highlights the complementary role of case reports in capturing adverse events for drugs not widely reported in spontaneous reporting systems.

SCARs are classified as delayed-type, T-cell-mediated type IV hypersensitivity responses, with their pathophysiological mechanisms yet to be fully understood. The median onset time of 21 days (range: 2–60 days) in our case review aligns with the delayed nature of type IV hypersensitivity reactions. In our case review, histopathological analysis of SCARs linked to anti-osteoporosis drugs revealed distinct patterns. Alendronate-associated acute localized exanthematous pustulosis showed neutrophil and eosinophil infiltration in the epidermis, with dermal edema and mild epidermal disruption, indicating a pustular reaction (Bautista-Villanueva et al., 2021). Clodronate-associated erythroderma displayed diffuse dermal inflammation with lymphocytes, histiocytes, and eosinophils, suggesting a hypersensitivity reaction affecting both skin layers (Pajus et al., 1993). Strontium ranelate-associated DRESS exhibited severe hypersensitivity features, including eosinophilic infiltration, epidermal spongiosis, keratinocyte necrosis, and basal layer degeneration, reflecting systemic immune activation (Kolitz et al., 2021; Drago et al., 2016; Jonville-Béra et al., 2009; Kinyó et al., 2011; Le Merlouette et al., 2011). Strontium ranelate-linked SJS revealed extensive epidermal necrosis, apoptosis, and subepidermal vesiculation, typical of SJS (Yang et al., 2014; Tan et al., 2011). Similarly, strontium ranelate-associated TEN showed full-thickness epidermal necrosis and dermo-epidermal separation with lymphocytic and eosinophilic infiltrates (Lee et al., 2009). Denosumab-associated AGEP featured robust neutrophilic inflammation, intracorneal pustules, parakeratosis, and mixed dermal infiltrates, consistent with AGEP’s profile (Marovt and Marko, 2024). In SJS/TEN, cytotoxic mediators such as granulysin and perforin from CD8^+^ T cells drive keratinocyte apoptosis and necrosis (Hasegawa and Abe, 2024). In DRESS and AGEP, Th2-driven cytokines (IL-4, IL-5, and IL-13) promote eosinophilic responses and systemic inflammation (Ramirez et al., 2023; Chen et al., 2023). Human leukocyte antigen (HLA) molecules likely present drug antigens, triggering T-cell activation, while cytokine dysregulation (IL-6, IL-8, and TNF-α) amplifies inflammation (Deschaseaux et al., 2011). Drug metabolism and genetic predispositions, such as HLA alleles, may enhance susceptibility by forming immunogenic complexes or reactive metabolites (Deshpande et al., 2021). Research has found that strontium ranelate-related SJS/TEN is significantly associated with HLA-A*33:03 and HLA-B*58:01 (Lee et al., 2016). The diverse histopathological and clinical presentations underscore the need for prompt drug withdrawal, anti-inflammatory therapies, and supportive care to mitigate severe outcomes, including secondary infections such as sepsis, which contribute to morbidity and mortality.

The systematic literature review included only 32 case reports (34 patients), which represents a significant limitation due to the small sample size. This restricted number of cases may not fully reflect the diversity of SCAR presentations or the broader clinical context of anti-osteoporosis drug-induced cutaneous reactions. The scarcity of published cases likely stems from the rarity of SCARs, underreporting, or limited recognition of these events in clinical practice (Hung et al., 2024), particularly for newer agents such as romosozumab. This limitation underscores the need for larger, prospective studies or registries to better characterize the incidence and clinical patterns of SCARs associated with anti-osteoporosis drugs. Furthermore, incomplete reporting of histopathological findings or standardized causality tools (e.g., the Naranjo scale) in some case reports may introduce uncertainty in attributing SCARs to specific anti-osteoporosis drugs.

The FAERS database is vital for post-market medication safety monitoring, identifying potential drug-related risks, including rare adverse events not detected in clinical trials. However, limitations such as reporting bias, underdocumentation, duplicate entries, and incomplete records, especially in older adults with multiple chronic conditions, hinder its effectiveness. These issues limit drug–drug interaction detection and the robustness of findings, particularly with few case reports. In our study, the limited number of case reports restricted the validation of rare adverse event signals as low reporting may reflect underdocumentation rather than true incidence. We integrated FAERS data with detailed case reports, combining FAERS’s broad, population-level signals with clinical case reports. This approach maximizes the reliability of our findings regarding rare events. However, FAERS signals indicate statistical associations, not causality, increasing the risk of false-positive results. These limitations highlight the need for cautious interpretation and validation through clinical studies or complementary data sources. In addition, our study focused on primary suspect drugs, excluding combination regimens. Future research should explore interactions between anti-osteoporosis drugs and concomitant medications.

## 5 Conclusion

In conclusion, this study provides the first comprehensive pharmacovigilance analysis of SCARs associated with anti-osteoporosis drugs, identifying significant signals for risedronic acid, zoledronic acid, and alendronic acid. These findings, supported by a systematic case review, highlight the need for heightened clinical vigilance, particularly in elderly female patients. Clinicians should assess patient-specific risk factors, such as HLA profiles and polypharmacy, before initiating therapy and monitor for cutaneous reactions during the first 2–8 weeks. Future research should focus on elucidating the immunopathogenic mechanisms of these reactions and evaluating the impact of combination therapies to further optimize patient safety.

## Data Availability

The original contributions presented in the study are included in the article/supplementary material; further inquiries can be directed to the corresponding author.
